# Earl Haig at Cardiff

**Published:** 1920-08-21

**Authors:** 


### Earl Haig at Cardiff.

On August 14 Earl Haig visited the Prince of Wales' Hospital for Limbless Sailors and Soldiers, Cardiff, in order to inspect the hospital and limb-shops. The Field-Marshal, who was accompanied by Lady Haig, was interested to watch the patients parade in the Rock Garden, where the paths are sloped at varying gradients from 1 in 12 to 1 in 3. This has proved one of the principal features of the hospital, and no patient is passed out until he is able to negotiate all the "hills." The result is that the percentage of satisfied patients is remarkably high, and men fitted in 1917 and 1918, when returning for replacements or repairs, have repeatedly acknowledged the value of the training they received when their first limbs were fitted.

After visiting the limb-shops, where over 1,700 artificial limbs had been made on the premises during the past three years, mainly by limbless men, Earl Haig inspected the patients and about twenty ex-patients assembled specially for the occasion. The veteran amongst these was Mr. John Chapman, wearing a long row of medals, including the Egyptian Medal and Star, the North-West Frontier of India Medal of 1897-8, with two clasps, the 1915 Star, the General Service and the Victory Medals for the Great War. Mr. Chapman was badly wounded at Gallipoli, and both legs had to be amputated. Earl Haig asked Mr. Chapman for details of his career, and with a hearty shake of his hand said, "I am proud of you."

### Treatment of Cripples in Wales.

An interesting development in the work of the hospital has been determined upon. Until recently the hospital has been fully occupied by officers, serving soldiers, and pensioners who lost limbs during the war. Now nearly all of the officers have been fitted with artificial limbs and the accommodation formerly occupied by them is therefore free. The Executive Committee, in re-erecting the buildings destroyed by fire in January last, has planned them so that in future the hospital can make and fit surgical boots and appliances for orthopædic cases, in the first place for disabled ex-Service men, as approved by the Ministry of Pensions. In the second place, the hospital will deal with South Wales civilian cripples, particularly children, who have in the past received treatment in London. This scheme has now received the confirmation of the Council of the hospital, and steps have been taken for fitting up the premises accordingly.

The accommodation set aside for cripples will be opened at a later date, and in the first instance some twelve or fourteen in-patients can be taken and a fair number of out-patients. This is special work that has not separately been dealt with in Cardiff. The present surgical staff includes Colonel Sir John Lynn-Thomas, K.B.E., C.B., C.M.G., Major S. Alwyn Smith, D.S.O., O.B.E., and Mr. James O. D. Wade, O.B.E.

